# Suicidal ideation and attempt among people living with HIV/AIDS in selected public hospitals: Central Ethiopia

**DOI:** 10.1186/s12991-021-00335-5

**Published:** 2021-02-19

**Authors:** Kefyalew Dagne Gizachew, Yigrem Ali Chekol, Elyas Admasu Basha, Shiferaw Abeway Mamuye, Abate Dargie Wubetu

**Affiliations:** 1 Department of Psychiatry, College of Health Sciences and Medicine, Debre Berhan University, P.O. Box 445, Debre Berhan, Ethiopia; 2 Department of Psychiatry, College of Health Sciences, Addis Ababa University, Addis Ababa, Ethiopia; 3 Department of Psychiatry, College of Health Sciences, Dilla University, Dilla, Ethiopia; 4 School of Nursing and Midwifery, Department of Pediatric and Child Health Nursing, College of Medicine and Health Sciences, Wollo University, Dessie, Ethiopia

**Keywords:** Suicidal ideation, Suicidal attempt, HIV/AIDS, Prevalence, Ethiopia

## Abstract

**Background:**

Suicide is the act of intentionally causing one's own death. HIV/AIDS continues to be associated with an under-recognized risk for suicidal behavior. Suicidal behavior among people living with HIV/AIDS is not only a predictor of future attempted suicide and completed suicide, but it is also associated with poor quality of life and poor adherence with antiretroviral therapy.

**Objective:**

The aim of this study was to assess the prevalence of suicidal ideation and attempt and associated factors among people living with HIV/AIDS in selected public hospitals of Amhara region, central Ethiopia.

**Methods:**

Institutional based cross-sectional study design was employed. The study was conducted in four public hospitals in North Shewa Zone from May to December 2017. Study population comprised all HIV-infected individuals from Antiretroviral Therapy (ART) clinic. A total of 348 study subjects were recruited using systematic random sampling and 326 completed the interview. Suicidality module from Composite International Diagnostic Interview (CIDI) was modified to assess suicidal behavior. Crude and adjusted OR was analyzed using logistic regression and the level of significance of association was determined at *P* value < 0.05.

**Result:**

A total of 326 (93.7%) study subjects were interviewed. The magnitude of suicidal ideation and attempt was found to be 16% and 7.1%, respectively. Low monthly income, living alone, suicidal thought before knowing seropositive status, family history of suicide, experiencing mild and moderate-to-severe depression and anxiety symptoms, being gossiped sometimes in the last 12 months of the study period due to HIV status and ever use of khat (a psychoactive substance) was statistically significant associated factor with suicidal ideation. And low monthly income, experiencing mild and moderate-to-severe depression and anxiety symptoms, being gossiped sometimes and often in the previous 12 months of the study period due to HIV status and using alcohol currently were significantly associated factors with suicidal attempt.

**Conclusion:**

Suicidal ideation and attempt among people living with HIV/AIDS (PLWHA) in this study were higher than prevalence in the general population. This shows suicidal ideation and attempt is a mental health concern of PLWHA and it needs great attention in Ethiopia.

## Background

Suicide is the act of intentionally causing one's own death or an intentional termination of one's own life, and it is often committed out of despair, the cause of which is frequently attributed to a mental disorder such as depression, bipolar disorder, schizophrenia, alcoholism, or drug abuse, and all these problems could be related with profound pain, hopelessness and despair that resulted in suicidal behavior [1, 2].

More than 800,000 people die by suicide annually, making it the 10th leading cause of death worldwide, and there are an estimated 10 to 20 million non-fatal attempted suicides every year [3, 4]. Developing and developed countries have parallel annual age standardized rates of suicide_11.2 and 12.7 per 100,000, respectively; however, 75% of suicide deaths globally is from developing countries [5].

Reports show that the rate of suicidality is higher in men than in women, with males three to four times more likely to kill themselves than females [4, 6], and suicidal attempts are more common in young people and females [3].

Studies from USA showed that, approximately 90% of completed suicides are associated with a mental disorder, and patterns are likely to be similar globally [3]. Factors related with suicide that are universal in both developed and developing world include youth or old age, low socioeconomic status, substance use, past history of suicide attempts, recent adverse life events, and access to methods to commit suicide [7].

Studies from sub-Saharan Africa (SSA) region show rates of suicide can be like those of developed countries and premature death from suicide has uncounted adverse consequences including: direct loss of life; loss of a breadwinner (parent or guardian); persistent psychological trauma for children, friends and relatives; and the loss of economic productivity for the nation at large [8].

Researchers have long known that human immunodeficiency virus (HIV) and AIDS (HIV/AIDS) is associated with an uncovered risk for suicidal behavior despite improvement in treatment and care that transformed HIV/AIDS into a treatable chronic illness [9]. Several factors predispose HIV/AIDS patients more likely to commit suicide including: they think that they will die earlier than they expected and worry that their deaths might be prolonged and imposes emotional and physical pain; and they may also be hopeless about the consequences of losing their jobs, their insurance, or their homes [10].

Suicidal behavior in HIV/AIDS is not only a predictor of future attempted suicide and completed suicide, but it is also associated with poor quality of life (QoL) and poor adherence with antiretroviral therapy [11, 12]. Existing studies report that important predictors for poor QoL among HIV-infected persons are: physical manifestations of the disease, antiretroviral therapy, psychological well-being, social support systems, coping strategies, spiritual well-being, and psychiatric comorbidities and these all could lead to suicidal behavior [13].

Reports shows that HIV has been associated with increased thoughts of suicidality, but estimates of the prevalence of lifetime suicidal ideation and attempt, and risks for new-onset suicide among HIV-infected individuals are not widely available in the era of modern combined antiretroviral treatment (cART) [14]. Findings also suggest that risk of suicidality is higher during early times (initial weeks) after positive test for HIV and then decrease as people infected with HIV start to adjust to their HIV status [15, 16].

Studies conducted before the introduction of HIV drugs_highly active antiretroviral therapy (HAART) showed an increased risk of completed suicide in patients with HIV/AIDS; that was 7 to 36 times greater than in the non-HIV-infected population [17, 18]. Since the introduction of HAART, findings suggests that suicidality among HIV-infected individuals decreased significantly, but remain above the rate reported in the general population [19]; and it may be mediated more often by factors other than HIV/AIDS itself including: depressive symptoms, alcohol use disorder, or other substance use disorders [20].

Risk of suicidality among HIV-infected patients may be higher than in populations with other chronic medical illnesses, such as cancer [21]. Suicidal ideation, attempted suicide, and committed suicide occur at a higher rate among patients with HIV infection than in the general population and individuals with HIV/AIDS are subject to disease-specific stressors and to a greater number of general suicide risk factors [14, 22].

To compare rate of suicidality between HIV-infected individuals and other patients with medical, mental conditions or the general public, the following were mentioned as instances. Suicidal behavior among cohorts of severe mental illness patients in rural Ethiopia was reported as; cumulative risk of suicide attempt, 26.3% for major depression; 23.8% for bipolar I disorder; 13.1% for schizophrenia [23]. Another study at University of Gondar Hospital Psychiatric Clinic patients estimated a 19.2% attempted suicide at least once after the onset of mental illness and 64.8% had suicidal ideation [24].

But reports on suicidal study in Ethiopia using the general population showed the lifetime suicide attempt of 3.2% at Butajira; and the prevalence of current suicidal ideation was 2.7%, and lifetime prevalence of suicidal attempt was 0.9% at Addis Ababa in which hanging and poisoning were the commonest methods of attempting suicide [25, 26]. A more recent comparative study at Northwest Ethiopia showed the prevalence of suicidal behavior in people with epilepsy to be 18.2%, and in the community sample to be 9.8% [27].

Previous studies in Ethiopia reported different prevalence of suicidal behavior among people living with HIV/AIDS (PLWHA), findings reported as follows. Prevalence of suicidal ideation and attempt among PLWHA were reported to be 22.5% and 13.9% at Zewditu Memorial Hospital, Addis Ababa [28]; 33.6% and 20.1%, at Debark Hospital, North Gondar [29]; and 24.3% and 12.6% at Hiwot Fana Specialized Hospital, Harar, respectively [30].

As per our search of data bases, reports from sub-Saharan African countries including Ethiopia are few on suicidality and HIV/AIDS, and screening suicidal behaviour is a highly recommended strategy to prevent mental health related deaths [31]. But the association between suicidality and HIV infection is attracting attention to date [32]. The rapidly growing literature on HIV/AIDS in Ethiopia has failed to examine the prevalence, associated factors, and its impacts of suicidality among people living with HIV/AIDS. It is therefore anticipated that this study findings will contribute to the development of local knowledge about suicidal behavior and HIV/AIDS and be used to scale-up the assessment of suicide risk in these patients involving a thorough exploration of these vulnerabilities.

### Objective

The aim of this study was to assess prevalence and factors associated with suicidal ideation and attempt among people living with HIV/AIDS in selected public hospitals, in central Ethiopia.

## Methods

### Study design, period, and area

Institutional based cross-sectional study design was employed from May to December 2017. The study was conducted in selected public hospitals of North Shewa Zone, which provides ART and pre-ART HIV/AIDS treatment and services. North Shewa Zone is one of the 11 Zones in Amhara administrative regional state and the City is Debre Berhan town, located 695 km from Bahir-Dar, capital of the region and 130 km from Addis Ababa, capital of Ethiopia. There are seven public hospitals in North Shewa Zone, one referral hospital and six primary hospitals. During data collection period, only three primary hospitals and the referral hospital were giving comprehensive HIV care and treatment and all are included in the study. Debre Berhan Comprehensive Specialized Hospital is the only referral hospital not only in the town but also in that zonal administrative catchment area (North Shewa Zone) and provides services for people from some part of Afar Region and it is estimated it serves for more than two million people. According to North Shewa zonal health office report of 2017 for comprehensive pre-ART and ART services (HIV/AIDS care) in all health facilities, these selected hospitals provide for the total number of 3,406 HIV-positive adults receiving clinical care during the data collection period: Debre Berhan Comprehensive Specialized Hospital, 2038 (95 on pre-ART and 1943 on ART); Ataye Primary Hospital, 291 (41 on pre-ART and 198 on ART); Alem ketema Enat Hospital, 690 (50 on pre-ART and 640 on ART); Mehal Meda Hospital, 439 (23 on pre-ART and 640 on ART).

### Source population

All HIV-infected patients receiving treatment of ART at ART clinics in public hospitals of North Shewa Zone in Amhara region, Ethiopia.

#### Study population

All HIV infected patients receiving treatment of ART at ART clinics in selected public hospitals of North Shewa Zone and who were available during the study period.

#### Inclusion criteria

The sample includes participants aged 18 years and above, tested HIV positive and currently receiving care and treatment for HIV/AIDS in all selected public hospitals providing ART services.

#### Exclusion criteria

Those with severe illness and unable to communicate for data collection.

### Sample size determination

In this study, sample size was determined using single population proportion formula. Previous prevalence of suicidal ideation (34.7%) in a study conducted in Benin City, Nigeria, was taken to obtain the maximum sample size at 95% certainty with a maximum discrepancy of 5% between the sample and the underlying population. For possible none response during the survey, the final sample size was increased by 10% and determined to be 348.

### Sampling procedures

Systematic random sampling technique was employed to recruit participants from each of the four sites. Proportional allocation was used among all public hospitals which has been providing HIV/AIDS care services. Sampling fractions was determined by dividing total study population who has monthly follow up during study period (pre-ART and ART clients) by the total sample size and participants were interviewed every 10th interval (see Additional file 1).

### Data collection instruments and operational definitions

A semi-structured questionnaire was used to collect data on socio-demographic characteristics (age, sex, ethnicity, religion, education, occupation, and marital status).

Past psychiatric history, suicidal history (suicidal attempts or expression of thoughts) before HIV infection, family history of suicide (attempted or completed suicide) and family history of known psychiatric illness was measured with participants’ self-report. Productive work (employment status) was defined as any work without monetary benefit and constitutes household work, studying or other work without payment [33].

Suicidality module from Composite International Diagnostic Interview_CIDI [34] was modified for our study context to assess suicidal behavior among people living with HIV/AIDS. CIDI was adapted in to Amharic language and determined to be reliable, acceptable, and feasible in Ethiopian mental health studies [35]. Suicidal ideation was defined as if the respondent answers “Yes” for the question “have you ever seriously thought about committing suicide after you know your sero-status?” Suicidal attempt was defined as if the respondent answers “Yes” for the question “have you ever attempted suicide after you know your sero-status?”.

Presence of co-morbid diseases were defined when subjects have at least one or more self-reported known chronic medical or physical diseases. A recent CD4 count, HIV/AIDS stage and duration since HIV status known were taken from respective formats and patients’ chart.

Substance use was assessed using purposely specified substances from "The alcohol, smoking and substance involvement screening test, ASSIST” [36]. ASSIST was developed for use in different cultural context and psychometric properties were tested using data across countries (both developing and developed) and recommended to be reliable, valid and simple for administration [37]. Ever use of substance was defined as ever use of at least one of specified substances in life time; and current use of substance was defined as use of at least one of specified substances in the previous three months before data collection.

Presence of anxiety and depression together and independently were identified using an ultra-brief screening scale for anxiety and depression: the PHQ-4 (the Four-Item Patient Health Questionnaire for Anxiety and Depression) [38]. PHQ-4 is a valid ultra-brief tool for detecting both anxiety and depressive disorders and it is shown to be excellent screening tool [38], and translated in to Amharic in this study. A cut point of ≥ 3 on for PHQ–2 scale has a sensitivity of 83% and specificity of 90% for major depressive disorder [39]; and cut point of ≥ 3 for GAD–2 scale has sensitivity of 88% for generalized anxiety disorder [40]. Combined anxiety and depression symptoms was defined positive from the total sum of PHQ-4 scores, and rated as normal (0–2), mild (3–5), moderate (6–8), and severe (38). Presence of anxiety symptoms alone was suggested by total score of ≥ 3 for first 2 questions of PHQ-4 [38], and depressive symptoms suggested by total score of ≥ 3 for last 2 questions of PHQ-4 [38].

Perceived social support was assessed using Oslo-3 item Social Support (OSS-3) scale to see the degree of social support and this scale is widely used in Ethiopia [41, 42]. OSS-3 in this study was scored according to total points ranging from 3–14; “poor support” 3–8, “moderate support” 9–11, and “strong support” 12–14 [41].

FICA Tool for Spiritual Assessment which includes Faith or belief, Importance of spirituality, individual’s spiritual Community, and interventions to Address spiritual needs was applied to evaluate the strength of spirituality, and FICA items were closely correlated with items from the quality of life (QoL) tools addressing spiritual component and this tool was feasible for clinical assessment of spirituality in previous studies [43]. Using FICA tool, a statement “Do you consider yourself spiritual or religious?”_ from “Faith, Belief, Meaning (F)” component was designed with “yes/no” option; and from “Importance and Influence (I)” component of FICA; “on a scale of 0 (not important) to 5 (very important), how would you rate the importance of faith/belief in your life?” was asked to subjects and mean score was considered as a cut off [43]. Experienced stigma was evaluated using two questions from “Illustrative questions by domain of HIV stigma and discrimination” for experienced stigma about gossip and exclusion from social gatherings or activities because of HIV status in the last 12 months [33].

Physical and sexual abuse was assed using Abuse Assessment Screen (AAS) with slight modification of timing, and AAS was shown to be a reliable and valid instrument for screening for abuse [44].

Current health status of PLWHA was assessed by asking participants to rate their current health status according to a five-category index: HIV-positive, with no symptoms, have symptoms, but have not had to change normal daily routines, have symptoms that have required to change parts of normal routines of daily activities (extra rest is not required during a normal day), because of symptoms, being in bed, or resting (less than half of waking hours), because of symptoms, being in bed, or resting (more than half of waking hours) [45].

### Data collectors and data quality control

The principal investigator recruited four-degree holder nurses (one from each of the four study sites) for data collection. Data collectors was trained and oriented on how to use of the questionnaire, the ethical principles of confidentiality and data management prior to their involvement with data collection. The questionnaire was designed and modified appropriately. The questionnaire including all instruments used were translated to local language_Amharic (participants interviewed with Amharic version) to be understood by all participants and translated back to English. Training was given for data collectors. Pre-test was done on 5% of the sample size before the start of actual data collection to test simplicity and easy understandability of the questionnaire at a distinct primary health care facility other than selected public hospitals for this study, and based on the finding from the pretest, the questioner was revised accordingly and time needed for interview was estimated. The data collectors were supervised routinely by assigned supervisors and the completed questionnaires was checked daily by the principal investigators and assistant investigators for completeness and consistency.

### Data processing and analysis

After data was checked for completeness and consistency, it was coded and entered in to Epi Data 3.1. Then, data was exported to SPSS version 20 for analysis.

In descriptive statistics; tables, graphs mean and frequency/percentage were used to present the information. Bivariate and multivariable logistic regression analysis was conducted to identify factors associated with suicidal ideation and attempt (independently) among persons living with HIV/AIDS. Only factors with *P*-value of ≤ 0.2 on bivariate analyses was kept for multivariable logistic regression analyses and 95% CI at *P*-value < 0.05 was considered as statistically significant.

The standard method of entry (enter method) was used to select variables. Collinearity diagnostics for continuous variables and Spearman rank correlation for categorical variables was performed and there was no multicollinearity or significant correlation between predictor variables. Model fitness was checked using Hosmer and Lemeshow Test, P-value of 0.31 and 0.57 for suicidal ideation and attempt, respectively.

### Ethical considerations

Ethical clearance was obtained from Debre Berhan University Institutional Review Board (IRB). Moreover, the investigator and data collectors have strived to protect and respect the privacy, secrecy, and wellbeing of participants with these conditions. The data collected for the purpose of this study never contained identifying information, thus ensuring the secrecy of the participants. All the data collected was used for the purpose of this study only. Participants was fully informed about the aims and methods of the study prior to starting the interview and informed written consent was obtained.

## Result

A total of 348 individuals living with HIV/AIDS were planned to participate in this study and out of which 326 (93.7%) participants completed the interview. A total of 22 non-respondent participants were due to; refusals to participate (13), and incomplete data (09).

### Socio-demographic characteristics

The mean age of respondents was 39.2 (Standard deviation, SD = 10.0). From the total study subjects, 326 (42.9%) were married and 59.8% were female in gender. Majority, (92%) of the participants were Orthodox Christian in religion. Almost 86% of the participants were living in an urban area; and 232 (71.2%) were living with their family. Few of the respondents (0.9%) who are living with HIV/AIDS were from prison. One hundred eight (33.1%) participants attended primary education. Almost 29% of individuals (first quartile) earn less than 300 Ethiopian Birr (ETB). In this study, 63 (19.3%) participants experienced hunger at least once because of lack of food in the previous month (Table 1).

**Table 1:** Distribution of PLWHA by socio-demographic factors at selected public hospitals, North Shewa Zone, Amhara Region, Ethiopia, 2017 (*n* = 326)

Variables	Frequency	Percentage (%)
Age (in years)		
18–27	28	8.6
28–37	121	37.1
38–47	104	31.9
> = 48	73	22.4
Mean age (± SD)	39.20 ± 10.0	
Sex		
Male	131	40.2
Female	195	59.8
Religion*		
Orthodox	300	92.0
Others	26	8.0
Marital status		
Married	140	42.9
Never married	59	18.1
Divorced	81	24.8
Widowed	46	14.1
Ethnicity*		
Amhara	300	92.0
Oromo	22	6.7
Others	4	1.2
Educational status		
Informal education	108	33.1
Primary education	108	33.1
Secondary education	64	19.6
Tertiary education	46	14.1
Employment status		
Unemployed/none	27	8.3
Productive	58	17.8
Income generating	241	73.9
Place of residence		
Urban	280	85.9
Rural	46	14.1
Average monthly income		
< 300 ETB	94	28.8
300–879 ETB	69	21.2
880–1800 ETB	85	26.1
> 1800 ETB	78	23.9
Hunger in the last month		
Yes	63	19.3
No	263	80.7
Current living status		
Living with family	245	75.2
Living alone	81	24.8

^*^Others (religion = Muslim, Protestant, Catholic; Ethnicity = Afar, Gurage, Tigre)

### Clinical and HIV-related factors

The clinical characteristics of the study participants is shown in (Table 2). Of the total individuals living with HIV/AIDS, 77.9% have at least one child; and 12.6% of these children are living with HIV/AIDS. Partners of participants (disclosure status of partner) who were living with HIV constitute 39% and the same proportion (39.3%) have unknown HIV status. Almost 90% of responders were in clinical stage1 of HIV/AIDS. Nearly all individuals started HAART; 312 (95.7%) and 34 (10.4%) who had started HAART were discontinued their ARV medication in a duration of treatment at least once. Off all the respondents; 60 (18.4%) reported that they have known medical illness; and the majority, 27 (8.3%) was comorbid mycobacterium infection. Few of the subjects, (10.7%) had CD4 count less than 200 cells/µl.

**Table 2:** Distribution of PLWHA by clinical factors at selected public hospitals, North Shewa Zone, Amhara Region, Ethiopia, 2017 (*n* = 326)

Variables		Frequency	Percentage (%)	
Past psychiatric illness				
Yes		24	7.4	
No		302	92.6	
Family history of psychiatric illness				
Yes		11	3.4	
No		315	96.6	
History of suicidal thoughts before sero-status known				
Yes		22	6.7	
No		304	93.3	
History of suicidal attempt before sero-status known				
Yes		18	5.5	
No		308	94.5	
Family history of suicidality				
Yes		13	4.0	
No		313	96.0	
Symptoms of anxiety and depression				
Normal		244	74.8	
Mild		40	12.3	
Moderate to severe		42	12.9	
History of known medical/psychical illness				
Yes		60	18.4	
No		266	81.6	
Time elapsed since sero-status known				
< 4 years		98	30.1	
4–7 years		87	26.7	
7–9 years		67	20.6	
> 9 years		74	22.7	
HIV staging				
Stage 1		292	89.6	
Stage 2		21	6.4	
Stage 3		13	4.0	
CD4 count (cells/µl)				
< 200		35	10.7	
200–499		149	45.7	
> 500		142	43.6	
Started taking ARV drug				
Yes		312	95.7	
No		14	4.3	
Ever discontinued ARV drugs				
Yes		34	10.4	
No		292	89.6	
HIV status of partner (disclosure status of partner)				
Positive		127	39.0	
Negative		71	21.8	
Unknown		128	39.3	
Have a child				
Yes		254	77.9	
No		72	22.1	
Have child with HIV				
Yes		41	12.6	
No		285	87.4	
Disclosure of HIV status				
Yes		293	89.9	
No		33	10.1	
Have family member with HIV				
Yes		112	34.4	
No		214	65.6	
Self-rated current health status				
HIV positive with no symptoms		251	77.0	
Have symptoms, not changed normal daily routines		45	13.8	
Symptoms, change normal daily routines; extra rest not required		22	6.7	
Symptoms, in bed, or resting, less than half of waking hours		8	2.5	

From a total of 326 study participants; 24 (7.4%) reported that they have previously diagnosed psychiatric illness. Mild comorbid depression and anxiety was reported as 12.3% and moderate-to-severe depression and anxiety to be 12.9%. And, the study suggests 13.8% and 12.6% of the respondents have independent depression and anxiety, respectively. From the total participants of the study, 22 (6.7%) had thought to kill themselves before they had known their HIV/AIDS status of positivity; and of them 18 (5.5%) attempted suicide after the suicidal idea.

Participants self-rated their current health status and 251 (77%) reported as having HIV with no symptoms. Two hundred twenty-eight (70%) study subjects were found to be in a duration of 5 years and above since diagnosis for HIV. Almost 34.5% of respondents have family member with HIV infection. A total of 293 (89.9%) participants disclosed their HIV status to significant others; and of them 213 (65.3%) choose their parent (father or mother) who should be told about their personal decision of disclosing. Only 133 (40.8%) of married individuals have got balance of honesty from their spouse to hear their HIV status disclosure.

### Psychosocial factors

From the total individuals included in the study (326), 265 (81.3%) considered oneself as spiritual and 69.9% rate the importance of being spiritual as important. Less than half of participants (45.4%) have perceived and labeled under having moderate-to-strong social support (Table 3).

**Table 3:** Distribution of PLWHA by psychosocial factors and social support at selected public hospitals, North Shewa Zone, Amhara Region, Ethiopia, 2017 (*n* = 326)

Variables	Frequency	Percentage (%)
Physical abuse since sero-status known (hit, slapped, kicked, or physically hurt someone?)		
Yes	15	4.6
No	311	95.4
Ever history of forced sex		
Yes	7	2.1
No	319	97.9
Social support		
Poor support	178	54.6
Moderate-to-strong support	148	45.4
History of gossip with the last 12 months because of HIV status		
Often	35	10.7
Sometimes	67	20.6
Hardly ever	32	9.8
Never	192	58.9
Excluded from social gatherings or activities in the last 12 months		
Often	41	12.6
Sometimes	64	19.6
Hardly ever	33	10.1
Never	188	57.7
Consider oneself spiritual or religious		
Yes	265	81.3
No	61	18.7
Rate of importance of faith/belief in life (mean score of scale from 0 to 5)		
Important (> = 3.96)	228	69.9
Not important (< 3.96)	98	30.1

In the dimension of physical and sexual abuse, 15 (4.6%) have been hit, slapped, kicked, or otherwise physically hurt by someone since they are found to have seropositive status disclosure; 7 (2.1%) also had history of forced sexual intercourse in life time. In the stigma domain, 192 (58.9%) subjects were never aware of being gossiped because of their HIV status (Table 3).

### Substance use characteristics of the respondents

From the total individuals involved in the study, 6.1%, 45.7% and 11.3% use tobacco, alcohol and khat at least once in their life time, respectively. Among ever users, current use (last 3 months use) was three (15.0%), 97 (65.1%) and 9 (24.3%) for tobacco, alcohol and khat, respectively (Table 4).

**Table 4:** Distribution of PLWHA by substance use characteristics at selected public hospitals, North Shewa Zone, Amhara Region, Ethiopia, 2017 (*n* = 326)

Variables	Frequency	Percentage (%)
Ever use of tobacco		
Yes	20	6.1
No	306	93.9
Ever use alcohol		
Yes	149	45.7
No	177	54.3
Ever use khat		
Yes	37	11.3
No	289	88.7
Current use of tobacco		
Yes	3	0.9
No	323	99.1
Current use alcohol		
Yes	97	29.8
No	229	70.2
Current use khat		
Yes	9	2.8
No	317	97.2

### Prevalence of suicidal ideation and attempt

**Fig. 1:**
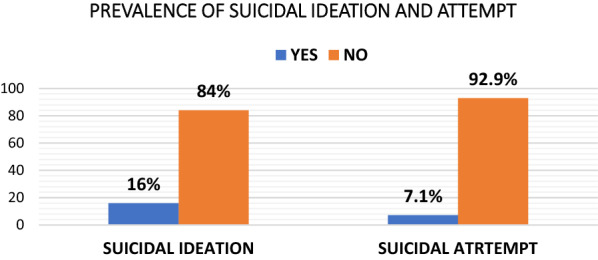
Prevalence of suicidal ideation and attempt at selected public hospitals, North Shewa Zone, Amhara Region, Ethiopia, 2017 (*n* = 326)

Among the total of 326 study subjects, those who replied “Yes” for suicidal ideation and attempt questions was considered having suicidal idea and attempt. Therefore, the overall prevalence of suicidal ideation and attempt among HIV/AIDS patients after knowing their seropositive status was 16% (95% CI 12.3, 19.9) and 7.1% (95% CI 4.6, 9.8), respectively (Fig. 1).

From the overall prevalence of suicidal attempt (7.1%), 34.8% of participants attempted suicide in the last month of the study period, and the majority (73.9%) used poisoning as a method of suicidal attempt. The primarily held reason for suicidal attempt was fear of the consequence of HIV, i.e., physical illness and stigma (60.9%). Among 23 individuals who attempted suicide since HIV status known, eight (34.8%) attempted suicide within 1 month of knowing HIV seropositive status (Table 5).

**Table 5:** Summary table for suicidal ideation and attempt characteristics at selected public hospitals, North Shewa Zone, Amhara Region, Ethiopia, 2017

Variables	Frequency	Percentage (%)
Suicidal ideation (*n* = 326)		
Yes	52	16.0
No	274	84.0
Duration of suicidal thought since HIV status known (*n* = 52)		
Within a month (< = 1 month)	34	65.4
1–3 months	12	23.1
More than 3 months (> = 3 months)	6	11.5
Suicidal idea with in the last one month (*n* = 52)		
Yes	15	28.8
No	37	71.2
Suicidal plan after HIV status known (*n* = 52)		
Yes	31	59.6
No	21	40.4
Suicidal attempt (*n* = 326)		
Yes	23	7.1
No	303	92.9
Planned suicidal attempt (*n* = 23)		
Yes	21	91.3
No	2	8.7
Attempted suicide among planned suicide (*n* = 31)		
Yes	21	67.7
No	10	32.3
Duration of suicidal attempt since HIV status known (*n* = 23)		
Within a month (< = 1 month)	18	78.3
1–3 months	2	8.7
More than 3 months (> = 3 months)	3	13.0
Suicidal attempt with in the last one month (*n* = 23)		
Yes	8	34.8
No	15	65.2
Frequency of suicidal attempt (*n* = 23)		
One attempt	13	56.5
Two attempts	4	17.4
Three or more attempts	6	26.1
Methods used for attempted suicide (*n* = 23)		
Hanging	3	13.0
Poisoning	17	73.9
Others (jumping, electric shock)	3	13.0
Self-reported reason for attempted suicide (*n* = 23)		
Poverty	5	21.7
Fear of HIV (physical illness, stigma)	14	60.9
Other reasons/combinations	4	17.4
Response most describes the seriousness of the suicidal attempt (*n* = 23)		
Serious attempt to kill oneself and it was only luck that didn’t succeed	7	30.4
Tried to kill oneself, but the method was not fool-proof	13	56.5
Attempt was cry for help, and did not intend to die	3	13.0

### Factors associated with suicidal ideation and attempt

Low monthly income, living alone, suicidal thought before knowing seropositive status, family history of suicide, experiencing mild and moderate-to-severe depression and anxiety symptoms, being gossiped sometimes in the last 12 months of the study period due to HIV status and ever use of khat (a psychoactive substance) was statistically associated factors with suicidal ideation (Table 6). And low monthly income, experiencing mild and moderate-to-severe depression and anxiety symptoms, being gossiped sometimes and often in the previous 12 months of the study period due to HIV status and using alcohol currently were factors independently associated with suicidal attempt (Table 7).

**Table 6:** Bivariate and multivariable logistic regression analysis for factors associated with suicidal ideation among PLWHA at selected public hospitals, North Shewa Zone, Ethiopia, 2017 (*n* = 326)

Variables	Suicidal ideation		COR (95% CI)	AOR (95% CI)
	Yes	No		
Age (in years)				
18–27	4	24	1.00	1.00
28–37	24	97	1.49 (0.47, 4.68)	2.30 (0.36, 14.50)
38–47	18	86	1.26 (0.39, 4.06)	2.13 (0.32, 14.03)
> = 48	6	67	0.54 (0.14, 2.07)	0.55 (0.06, 4.74)
Marital status				
Married	16	124	1.00	1.00
Never married	11	48	1.78 (0.77, 4.10)	1.68 (0.46, 6.14)
Divorced	18	63	2.21 (1.06, 4.63)	2.03 (0.64, 6.46)
Widowed	7	39	1.39 (0.53, 3.63)	0.61 (0.13, 2.87)
Ethnicity				
Amhara	45	255	0.18 (0.02, 1.29)	0.13 (0.002, 10.77)
Oromo	5	17	0.29 (0.03, 2.65)	0.09 (0.001, 9.84)
Others	2	2	1.00	1.00
Employment status				
Unemployed/none	8	19	2.25 (0.92, 5.51)	1.01 (0.20, 5.09)
Productive	6	52	0.62 (0.25, 1.54)	0.43 (0.12, 1.52)
Income generating	38	203	1.00	1.00
average monthly income				
< 300 ETB	23	71	4.73 (1.70, 13.13)	4.02 (1.20, 3.51) *
300–879 ETB	11	58	2.77 (0.91, 8.42)	1.87 (0.52, 6.72)
880–1800 ETB	13	72	2.64 (0.89, 7.78)	1.44 (0.41, 5.04)
> 1800 ETB	5	73	1.00	1.00
Hunger in the last month				
Yes	17	46	2.41 (1.24, 4.66)	0.64 (0.20, 2.07)
No	35	228	1.00	1.00
Current living status				
Living with family	32	213	1.00	1.00
Living alone	20	61	2.18 (1.17, 4.09)	2.46 (1.05, 5.74) *
Past psychiatric illness				
Yes	7	17	2.35 (0.92, 5.99)	0.58 (0.11, 3.05)
No	45	257	1.00	1.00
History of suicidal thoughts before HIV status known				
Yes	15	7	15.46 (5.92, 40.42)	4.33 (0.84, 22.36)
No	37	267	1.00	1.00
History of suicidal attempt before HIV status known				
Yes	15	3	36.62 (10.12, 132.55	8.74 (1.30, 58.88) *
No	37	271	1.00	1.00
Family history of suicidality				
Yes	8	5	9.78 (3.06, 31.26)	5.79 (1.34, 25.08) *
No	44	269	1.00	1.00
Started taking ARV drug				
Yes	48	264	1.00	1.00
No	4	10	2.20 (0.66, 7.30)	1.42 (0.25, 8.09)
Self-rated current health status				
HIV positive with no symptoms	31	220	1.00	1.00
Have symptoms, not changed normal daily routines	16	29	3.92 (1.91, 8.02)	2.10 (0.81, 5.48)
Symptoms, change normal daily routines; extra rest not required	4	18	1.58 (0.50, 4.97)	0.24 (0.04, 1.36)
Symptoms, in bed, or resting, less than half of waking hours	1	7	1.01 (0.12, 8.52)	0.08 (0.002, 3.03)
Symptoms of anxiety and depression				
Normal	23	221	1.00	1.00
Mild	13	27	4.63 (2.10, 10.18)	5.78 (2.13, 15.75) ***
Moderate to severe	16	26	5.91 (2.78, 12.60)	5.47 (2.01, 14.88) ***
Social support				
Poor support	34	144	1.71 (0.92, 3.17)	1.08 (0.42, 2.75)
Moderate-to-strong support	52	274	1.00	1.00
History of gossip with the last 12 months because of HIV status				
Often	11	24	4.43 (1.87, 10.50)	2.38 (0.60, 9.50)
Sometimes	16	51	3.03 (1.44, 6.37)	3.26 (1.07, 9.97) *
Hardly ever	7	25	2.71 (1.03, 7.13)	1.36 (0.31, 6.02)
Never	18	174	1.00	1.00
Consider oneself spiritual or religious				
Yes	37	228	1.00	1.00
No	15	46	2.01 (1.02, 3.96)	1.17 (0.36, 3.84)
Rate of importance of faith/belief in life (mean score of scale from 0 to 5)				
Important (> = 3.96)	32	196	1.00	1.00
Not important (< 3.96)	20	78	1.57 (0.85, 2.91)	1.25 (0.43, 3.70)
Ever use of tobacco				
Yes	6	14	2.42 (0.89, 6.63)	0.89 (0.17, 4.66)
No	46	260	1.00	1.00
Ever use of khat				
Yes	13	24	3.47 (1.63, 7.38)	5.58 (2.08, 14.98) ***
No	52	274	1.00	1.00
Current use of khat				
Yes	3	6	2.74 (0.66, 11.30)	0.51 (0.05, 4.75)
No	49	268	1.00	1.00

*P value is significant at *P* < 0.05 **P value is significant at *P* < 0.01, ***P value is significant at *P* ≤ 0.001, 1.00 = reference for category, *P* value of Hosmer and Lemeshow test = 0.31

**Table 7:** Bivariate and multivariable logistic regression analysis for factors associated with suicidal attempt among PLWHA at selected public hospitals, North Shewa Zone, Ethiopia, 2017 (*n* = 326)

Variables	Suicidal attempt		COR (95% CI)	AOR (95% CI)
	Yes	No		
Marital status				
Married	6	134	1.00	1.00
Never married	5	54	1.78 (0.77, 4.10)	2.60 (0.55, 12.24)
Divorced	10	71	2.21 (1.06, 4.63)	2.42 (0.52, 11.32)
Widowed	2	44	1.39 (0.53, 3.63)	0.58 (0.08, 4.20)
Employment status				
Unemployed/none	5	22	3.42 (1.14, 10.32)	1.66 (0.25, 11.11)
Productive	3	55	0.82 (0.23, 2.94)	0.46 (0.10, 2.15)
Income generating	15	226	1.00	1.00
Place of residence				
Urban	22	258	1.00	1.00
Rural	1	45	0.26 (0.03,1.98)	0.19 (0.02, 1.72)
Average monthly income				
< 300 ETB	10	84	9.17 (1.15, 73.28)	11.55 (1.12, 18.86) *
300–879 ETB	3	66	3.50 (0.36, 34.46)	3.57 (0.28, 45.98)
880–1800 ETB	9	76	9.12 (0.13, 73.73)	9.21 (0.92, 91.84)
> 1800 ETB	1	77	1.00	1.00
Hunger in the last month				
Yes	9	54	2.96 (1.22, 7.20)	1.64 (0.45, 5.96)
No	14	249	1.00	1.00
Past psychiatric illness				
Yes	5	19	4.15 (1.39, 12.40)	2.893 (0.573, 14.614)
No	18	284	1.00	1.00
Family history of suicidality				
Yes	3	10	4.40 (1.12, 7.25)	5.099 (0.80, 32.499)
No	20	293	1.00	1.00
Self-rated current health status				
HIV positive with no symptoms	12	239	1.00	1.00
Have symptoms, not changed normal daily routines	8	37	4.31 (1.65, 11.24)	1.37 (0.35, 5.33)
Symptoms, change normal daily routines; extra rest not required	2	20	1.99 (0.42, 9.53)	0.43 (0.05, 3.98)
Symptoms, in bed, or resting, less than half of waking hours	1	7	2.85 (0.32, 25.02)	0.17 (0.01, 5.74)
Symptoms of anxiety and depression				
Normal	8	236	1.00	1.00
Mild	7	33	6.26 (2.13, 18.39)	5.44 (1.64, 18.07) **
Moderate to severe	8	34	6.94 (2.44,19.71)	4.46 (1.40, 14.25) *
History of gossip with the last 12 months because of HIV status				
Often	7	28	6.61 (2.16, 20.26)	4.98 (1.38, 17.93) *
Sometimes	8	59	3.58 (1.25, 10.30)	5.14 (1.51, 17.52) **
Hardly ever	1	31	0.85 (0.10, 7.17)	0.77 (0.07, 8.01)
Never	7	185	1.00	1.00
Consider oneself spiritual or religious				
Yes	15	250	1.00	1.00
No	8	53	2.52 (1.02, 6.24)	2.76 (0.94, 8.13)
Rate of importance of faith/belief in life (mean score of scale from 0 to 5)				
Important (> = 3.96)	13	215	1.00	1.00
Not important (< 3.96)	10	88	1.88 (0.80, 4.45)	0.98 (0.27, 3.60)
Ever use of tobacco				
Yes	3	17	2.52 (0.68, 9.34)	1.51 (0.12, 19.65)
No	20	286	1.00	1.00
Current use of tobacco				
Yes	1	2	6.84 (0.60, 78.42)	3.78 (0.05, 285.73)
No	22	301	1.00	1.00
Current use of alcohol				
Yes	11	86	2.31 (0.98, 5.44)	3.34 (1.21, 9.25) *
No	12	217	1.00	1.00
Ever use of khat				
Yes	5	32	2.35 (0.82, 6.77)	2.05 (0.34, 12.52)
No	18	271	1.00	1.00

*P value is significant at *P* < 0.05, **P value is significant at *P* < 0.01, ***P value is significant at *P* ≤ 0.001, 1.00 = reference for category**,** P value of Hosmer and Lemeshow test = 0.57

## Discussion

### Prevalence of suicidal ideation and associated factors

The overall magnitude of suicidal ideation in this study was 16% (95% CI 12.3, 19.9). This result shows that significant proportion of people living with retroviral infection had a mental health problem of suicidal ideation. Hence, assessing suicidal ideation and giving appropriate intervention should become a substantial concern for professionals working at HIV/AIDS clinics.

The magnitude of suicidal ideation in this study was in line with the study done in HIV clinics of university of Washington and Alabama; 14% [9], US cities; 19% [46] and South Africa; 17.1% within 3 days after knowing HIV status [47]. The reason for the consistency of magnitude might be due to similar nature of the retroviral disease infection across regions such as: chronicity, decreased functioning (declining all dimensions of health), and the virus effect on the central nervous system (imposing cognitive decline) can lead to possible rational thinking loss, depression and other related mental disorders which in turn result in an increased vulnerability of PLWHA prone to thought about suicide.

The magnitude of suicidal ideation in this study was lower than those studies conducted in North America; 26% [14], Virginia Commonwealth University; 26% [48], Texas; 59% [49], South East London; 31% [22], Chelsea and London; 26.9% [32], Nigeria; Benin, and 34.7% [50]. The lower magnitude of suicidal ideation in this study might be due to the level of practical social support. As the fact that in developing countries including Ethiopia, there is strong social support and religion also has major role not to think about suicide. Increased level of understanding about nature and chronicity of the disease by HIV infected individuals, and presence (access and availability) of treatment for HIV/AIDS these days could also contribute for PLWHA to have a lower suicidal thought.

The magnitude of suicidal ideation in this study was higher than studies done using general population sample in Addis Ababa; 2.7% [26]. The difference might be due to difference in outcome of interest, literacy status, sample size, and study population. The study done at Addis Ababa aimed to current suicidal ideation and used urban representative sample (*N* = 10,203). Additionally, living in capital city may possibility increase the literacy rate of the study subjects; and probably may get information about how to live longer with retroviral disease by adhering to ARV drugs.

Thinking about suicide among PLWHA in this study was lower than a report on people having mental illness (64.8%), from a psychiatric clinic in Gondar Hospital [24]. Compared to previous studies in Ethiopia, our study reports a lower suicidal ideation prevalence: 22.5% at Zewditu Memorial Hospital, Addis Ababa [28]; 33.6% at Debark Hospital, North Gondar [29]; and 24.3% at Hiwot Fana Specialized Hospital, Harar [30]. This variation might be explained by the difference of study settings that our study mainly includes primary health care services which predominantly serves rural residents and under reporting is expected as a result of social desirability issue in fear of stigma, and lower literacy rate might cause limited awareness about psychological, social and physical impact of HIV/AIDS likely leading to reserved true responses of deicidal thoughts.

This study found that suicidal ideation was significantly associated among study subjects who have been gaining less than 300 Ethiopian Birr per month as compared with study subjects who earn greater than 1800 Ethiopian Birr. This result is consistent with the study conducted in New York City [51], US cities [46]. This finding might be due to that individuals who earn low monthly income which causes possible financial distress are unable to fulfill basic needs; have potential nutritional deficit and may impose external effect on participants emotional and psychological wellbeing.

Living alone was significantly associated with suicidal ideation as compared with individuals living with significant others and consistent with the study done in New York city [51], US cities [46], Puerto Rico [27] and South Africa [47]. The possible explanation for this consistency seems that being living with others is supportive and basic for emotional and social support; as supportive environment decreases the sense of isolation and not to think about suicide.

Having suicidal thoughts before knowing HIV status was statistically significant factor for current suicidal ideation. The association was in the same way of the studies done in semi-urban Uganda [11]. The association might be due to having previous suicidal thoughts increases the vulnerability of individuals to thought about suicidal after knowing the HIV status. Having family history of suicide was also significantly associated with suicidal ideation. This association might be possibly due to individuals who had suicide among their first-degree relatives are genetically prone for suicidality.

Study subjects who experienced mild and moderate-to-severe depression and anxiety symptoms had higher odds to have suicidal ideation as compared with individuals who had no symptoms. This finding is consistent with the finding of the studies done in New York City [51], Virginia Commonwealth University [48], Puerto Rico [52] and semi-urban Uganda [11]. The possible reason for the consistency might be the fact that individuals who suffer depression and anxiety symptoms are at higher risk of thinking about suicide due to hopelessness, worthlessness, and excessive and uncontrollable thoughts.

Ever use of khat (a psychoactive substance) is associated with suicidal ideation as compared with who did not use. This finding is in agreement with studies done in New York City [51], US cities [46] and Virginia Commonwealth University that substance or drug abuse (dependence) like intravenous drug use, cocaine use, and alcohol use were reported to be associated with suicidal ideation [48]. This consistency might be due to individuals who abuse psychoactive substances (like khat) are vulnerable to think about suicide due to declining health and the substance direct effect to the brain.

A psychosocial factor like being gossiped sometimes in the previous 12 months of study period due to the status of HIV positive were significantly associated for suicidal ideation as compared with individuals who did not witness gossip. This finding is in line with studies done in New York City [51]. The possible explanation for the association might be as a result of gossip for the status of HIV infection plays important role in thinking of suicide and feeling of stigmatized can make people more vulnerable for thinking suicidality.

### Prevalence of suicidal attempt and associated factors

The overall magnitude of suicidal attempt in this study was 7.1% (95% CI 4.6, 9.8). This result shows that significant proportion of people living with retroviral disease had attempted to kill themselves. Hence, assessing history of attempting suicide and giving immediate intervention should become a substantial concern for professionals working at HIV/AIDS clinics.

The magnitude of suicidal attempt in this study was in line with the study done in semi urban Uganda; 7.8% [11] and Nigeria (Benin); 9.3% [50]. This agreement in magnitude might be due to health literacy, weak interdepartmental communication, and tendency of high HIV infection-related stigmatization in African countries. Health literacy has important role for attempting suicide; as individuals had an information about the possibility of living longer with HIV, and attempting suicide would be less likely. The service fragmentation between HIV clinics and psychiatric clinics in African countries might have been played major role for the lack of screening suicide and giving psychological interventions. Stigma associated with HIV infection is higher in African countries [53] and the immediate outcome of stigma might be attempting suicide due to the feeling of isolation, loneliness and clouding of future life.

The magnitude of attempting suicide after knowing HIV status of positivity in the current study is lower than the studies done in North American medical centers; 13% [14], New York City; 26% [54], Texas; 50% [49], Puerto Rico; 22% [52], Chelsea and London; 20% [32]. This variation might be due to under reporting of suicidal attempt in fear of the social desirability issue of participants (disclosing suicidality might be considered as a personal weakness) in our study and the effect of religion, that suicide is considered as an additional sin in the view of some religious doctrines in our setting.

Studies using general population sample at Addis Ababa among 10,203 adults [26], and Butajira, Ethiopia [25] reported a lower prevalence of life time suicidal attempt; 0.9% and 3.2%, respectively, compared to our study finding. The difference in magnitude estimation might be due to the time of the study (far earlier in previous studies), sample size, study area and population of interest that general population comprises a mix of healthy, and mentally or medically ill individuals including HIC/AIDS. Like previous discussion on suicidal ideation section above, reported suicidal attempt in this study was lower compared to people with mental illnesses in rural Ethiopia [23] and psychiatric clinic at University of Gondar [24]. Similarly, compared to previous studies in Ethiopia, our study shows a lower suicidal attempt prevalence: 13.9% at Zewditu Memorial Hospital, Addis Ababa [28]; 20.1%, at Debark Hospital, North Gondar [29]; and 12.6% at Hiwot Fana Specialized Hospital, Harar [30].

This study found that suicidal attempt was higher among study subjects who have less than 300 ETB monthly income as compared with study subjects who gain greater than 1800 ETB. This result is consistent with the study conducted in New York City [51], US cities [46]. This finding might be because individuals who earn low monthly income (possible cause of financial distress) are unable to fulfill basic needs; have potential nutritional deficit, and impose external effect on participants emotional and psychological wellbeing.

Being gossiped (a psychological factor) sometimes and often in the last 12 months of the study period due to the status of HIV positivity were significantly associated for suicidal attempt as compared with individuals who didn’t witnessed gossip. This finding is in line with studies done in New York City [51]. Using alcohol in the last three months before the study period was significantly associated with suicidal attempt as compared with who did not use. This finding is in agreement with studies done in New York City [51], US cities [46] and Virginia Commonwealth University [48].

Among clinical factors that contributed for suicidal attempt was suffering from depressive and anxiety symptoms. Study subjects who experienced mild and moderate-to-severe depression and anxiety symptoms had higher odds to have suicidal attempt as compared with individuals who had no symptoms. This result is consistent with the finding of the studies done in New York City [20], Virginia Commonwealth University [48], Puerto Rico [52] and semi-urban Uganda [11].

## Limitations of the study

This study uses the most recent CD4 count by reviewing client’s chart; and not the actual CD4 count requested during data collection. There would be possibility of recall bias as participants might forget their suicidal experiences long ago in their life.

## Conclusion and recommendations

Prevalence of suicidal ideation and attempt among PLWHA in our selected hospitals were higher compared to previous reports of general population studies in Ethiopia. Low monthly income, living alone, suicidal thought before knowing seropositive status, family history of suicide, experiencing mild and moderate-to-severe depression and anxiety symptoms, being gossiped sometimes in the previous 12 months of the study period due to HIV status and ever use of khat were statistically significant associated factors with suicidal ideation. In addition, low monthly income, experiencing mild and moderate-to-severe depression and anxiety symptoms, being gossiped sometimes and often in the previous 12 months of the study period due to HIV status and using alcohol currently were significantly associated factors of suicidal attempt.

The following recommendations are suggested based our findings. It is better for North Shewa zonal health bureau to strengthen and scale up the primary health care (PHC) mental health program_Mental Health Gap Action Programme (mhGAP) which has been already started; and target should include PLWHA. And it is better to collaborate with regional health bureau and other stake holders to equip public hospitals with necessary mental health services and linkage with HIV/AIDS care clinics. In addition, it is better if interdepartmental communication between HIV and psychiatric clinics is initiated and make it functional and sustainable at Debre Berhan Comprehensive Specialized Hospital and another similar setting.

Besides, it is recommended to assess and address the social support system and economical aspect; previous self, or family history of suicidal behavior; concurrent depressive or anxiety symptoms, experience of stigma; and substance use (like khat and alcohol use) for HIV infected individuals.

Lastly, it is advised that future research should focus on recent risk assessment of suicidal behavior and actual committed suicide among PLWHA from HIV/AIDS care centers using more strong study designs (like follow-up study) comprising comparison groups which might help to develop or scale-up appropriate psychosocial support systems and interventions.

## Supplementary Information

**Additional file 1.** The schematic presentation of sampling procedures among PLWHA at selected hospitals of North Shewa Zone, Amhara region, Ethiopia, 2017.

## Data Availability

All relevant materials and data supporting the findings of this study are contained within the manuscript and relevant document will be available upon request.

## Abbreviations

ART: Antiretroviral therapy
ARV: Antiretroviral
BSI: Beck Scale for Suicide Ideation
CI: Confidence Interval
HAART: Highly active antiretroviral therapy
HIV/AIDS: Human Immune Deficiency Virus/Acquired Immune Deficiency Syndrome
OR: Odds ratio
PLWHA: People living with HIV/AIDS
PRO: Patient reported outcome
SPSS: Statistical Package for Social Science
SSA: Sub-Saharan Africa
UK: United Kingdom
USA: United States of America
WHO: World Health Organization
