# Supplementary material for: Suicidal ideation and attempt among people living with HIV/AIDS in selected public hospitals: Central Ethiopia
Source: Ann Gen Psychiatry. 2021 Feb 19;20:15. doi: 10.1186/s12991-021-00335-5 (PMC7896396; doi:10.1186/s12991-021-00335-5)
Supplement: Supplementary file 1 — Additional file 1. The schematic presentation of sampling procedures among PLWHA at selected hospitals of North Shewa Zone, Amhara region, Ethiopia, 2017. [file 12991_2021_335_MOESM1_ESM.docx]

**Additional Files**

### Additional file1: Sampling procedures

Total Sample Size (n=348)

PROPORTIONA ALLOCATION

HOSPITALS IN NORTH SHOA ZONE PROVIDING ART CARE

DEBRE BERHAN COMPREHENSIVE SPECIALIZED HOSPITAL (N1 = 2038)

ATAYE PRIMARY HOSPITAL

(N2 =239)

MEHAL MEDA PRIMARY HOSPITAL

(N4 = 439)

ENAT PRIMARY HOSPITAL

(N3= 690)

n1 = 208

n2 = 24

n3 = 71

n3 = 46

Additional file1. **The schematic presentation of sampling procedures among PLWHA at selected hospitals of North Shoa Zone, Amhara region, Ethiopia, 2017**
